# Multi-Level Protective Factors of Adolescent Smoking and Drinking

**DOI:** 10.3390/ejihpe13060071

**Published:** 2023-05-30

**Authors:** Réka Dudok, Bettina F. Piko

**Affiliations:** 1 Doctoral School of Education, University of Szeged, 6725 Szeged, Hungary; dudok.reka@edu.u-szeged.hu; 2 Department of Behavioural Sciences, University of Szeged, 6722 Szeged, Hungary

**Keywords:** smoking, drinking, adolescence, protective factors, self-control, school attachment, subjective well-being

## Abstract

Adolescence is the most critical life stage for experimentation with substance use; however, this is also the most suitable period for strengthening protective factors and thus promoting adult physical and mental health. Since smoking and drinking still appear among the most frequent types of substance abuse in Europe, this study aims to examine the role of potential protective factors at multiple levels for adolescent smoking and drinking: psychological factors at the individual level, aspects of school attachment at the school level, social support variables at the social level, and measures of quality of life at the level of mental health. This cross-sectional survey involved a sample of adolescents (aged 11–18 years, N = 276) in Budapest and villages in its metropolitan area (Hungary). In addition to descriptive statistics, logistic regression analyses were used to detect odds for potential protective factors. There were no sex differences in adolescents’ substance use. Self-control seems to be a universal and most determining protective factor against substance use, while other potential protective factors (self-esteem, resilience, social support from family or significant others, school attachment, and mental well-being) may also contribute to prevention. However, age and friend support acted as risk factors. Findings suggest that a complex approach to prevention should receive consideration.

## 1. Introduction

Smoking and alcohol consumption are the two most relevant contributing factors that cause death all over the world [1]. Adolescence is the most frequent period for trying these substances, and children who start substance use at this early stage have a greater chance of continuing these habits later, which may lead to severe physical and mental health consequences. Adolescence is the life period when involvement in risk-taking has long-term effects on a youth’s later health [2]. This is because during this critical transition phase of adolescence, rapid physical, emotional, cognitive, and social development allows adolescents to develop a range of health risk behaviors. On the other hand, this period may be the most optimal and appropriate time for laying the foundations for health promotion in adulthood. Thus, the importance of existing and further prevention and intervention targeting adolescent alcohol and tobacco-related factors remains a challenge. Previous studies have shown the complexity and multi-layered background of substance use and highlighted the interrelationship between individual, psychological, and social factors [3]. Therefore, a key to successful interventions and actions is a clear understanding of the development, predictors, and indicators of health risk behaviors.

Although traditional health risk behaviors, such as substance use, are slowly declining in the 21st century, these tendencies are not as prevalent in Hungary and Eastern Europe compared to Western countries [4]. The prevalence rates of smoking and alcohol use differ from highest to lowest in Eastern Europe, Southern Europe, Western Europe, and Northern Europe. Results from the Health Behaviour of School-aged Children study (HBSC) [5] and the European School Survey Project on Alcohol and Other Drugs (ESPAD Group) [6] showed substance use in Hungary appeared to be one of the most frequent health risk behaviors in Eastern Europe. Especially adolescents’ alcohol consumption is one of the highest health risks in Europe, and while boys drink more alcohol than girls, the rate of smoking among girls is higher than that of boys. In addition to smoking and drinking still having a considerable occurrence in Eastern Europe, they are closely connected to adolescents’ mental well-being and quality of life [7]. Therefore, we need to further explore and strengthen protective factors to support this declining tendency, including at the individual, school, and social levels. Furthermore, it would also be necessary to map differences in correlates of experimentation and regular use. 

Several individual-level protective factors may be identified in terms of adolescent smoking and drinking, such as self-esteem, self-control, coping, and resilience. 

Self-esteem is defined as an individual’s overall evaluation of their worth or value as a person and is often linked to feelings of confidence and self-respect [8]. Individuals with low self-esteem may be at higher risk of engaging in smoking and drinking as a way to cope with negative emotions or to fit in with social groups [9,10]. In relation to substance use, self-esteem was found to mediate the relationship between self-control and self-efficacy; therefore, it may be an asset in prevention [11]. Indeed, due to coping skills training in a substance abuse prevention program for adolescents, self-esteem was significantly improved as compared with a control group [12]. However, associations between measures of self-esteem and smoking or drinking are not consistent. Adolescent smoking is not necessarily related to self-esteem in adolescents [13], and sometimes people with higher self-esteem are more likely to be current drinkers [9]. This further emphasizes the importance of understanding the role of self-esteem in substance use.

Self-control has been defined as an individual’s ability to regulate their thoughts, emotions, and behaviors in order to achieve a desired goal [14]. Individuals with low self-control tend to be more impulsive, and they may lack the ability to delay gratification, which makes them more susceptible to engaging in smoking and drinking [15,16]. The self-control theory [17] suggests that when people experience stress or stressors, their ability to self-regulate their behaviors is diminished, and as a result, the likelihood of smoking and drinking increases. Consequently, those with weaker reflective processes and stronger impulsive drives may be at higher risk of engaging in smoking and drinking [18,19].

Coping refers to the conscious use of cognitive, affective, or behavioral efforts to manage or reduce the negative emotions or stress people experience in response to challenging situations [20]. The Tension Reduction Theory posits that alcohol and tobacco are consumed to achieve tension reduction [21,22]. Thus, substance use may often serve as a way of coping with stress [23]. Research results suggest that particularly proactive coping, which is a type of problem-focused coping, can play a role in reducing stress by assessing future goals and setting the stage to achieve them successfully [24]. In this way, it prepares an individual for potential future stressors. 

Resilience refers to an individual’s ability to adapt and recover from adversity or stress [25]. Resilience plays a role in smoking and drinking as it supports personal resistance and flexibility, helps adaptation in the face of stressors, and decreases the possibility of substance use [26]. Resilience may buffer the effects of stress on health behaviors so that resilient youth are better able to cope with stress and negative emotions, may be less likely to engage in substance use, and report better mental health [27].

At the school level, some important protective factors can also be found. Since children spend a lot of time in school, the school domain serves as a tool for secondary socialization, where children learn new behaviors and build connections with teachers and peers. While earlier studies focused mostly on negative experiences, such as problems with school achievement or adjustment in relation to substance use [28], recent studies have concentrated more on school climate, referring to interpersonal relationships, norms and values, social interactions, the school environment (physical, psychosocial, and earning), and both negative and positive experiences [29]. A positive school climate may act as an effective protective factor against dropout, bullying, aggressive and violent behavior [30], and substance use among adolescents [31]. School engagement or school attachment may particularly serve as a protective factor against problem behaviors [32].

Among the social protective factors, social relationships can significantly contribute to positive youth development [33]. Family, especially, may provide protection against adolescent substance use, such as smoking and drinking [34]. Familial protection can be explained partly by parental control and monitoring and partly by the good quality of the parent-adolescent relationship, which provides social support for children. However, not all types of social support can act as a protection; while family support usually decreases the likelihood of substance use [35], support from friends and peers appears to be risk factors for smoking and alcohol consumption [36].

Finally, several variables of quality of life may provide mental health protection against substance use, such as general or psychological well-being and satisfaction with life. Life satisfaction, as a global assessment of a person’s quality of life, reflects a positive attitude towards one’s life overall [37]. An earlier study revealed that cigarette smoking, regular alcohol use, binge drinking, and illicit drug and steroid use were all significantly associated with reduced life satisfaction; in addition, this was also the case in terms of the first use of cigarettes and alcohol [38]. An association between psychological well-being and substance abuse among South African adolescents was also concluded [39]. Data from the 2017–2018 Health Behaviours in School-aged Children study showed that adolescents’ mental well-being was closely connected to substance use and their level of social support [7].

Based on the literature, we aimed to examine the role of potential protective factors in adolescent smoking and drinking at multiple levels: psychological factors at the individual level, school attachment factors at the school level, social support variables at the social level, and measures of quality of life at the level of mental health. First, descriptive statistics and sex differences in substance use and protective factors were examined. In this case, we hypothesized a lack of sex differences in substance use or a slightly higher incidence rate among girls, particularly in smoking [6]. Subsequently, we calculated correlation coefficients for the scales, assuming strong interrelationships between them. Then, using logistic regression analyses, we determined their contribution to differences in the odds of adolescents’ smoking and drinking. We assumed that each of the potential protective factors (except for social support from friends) might be a significant predictor of smoking and drinking, particularly in terms of current use. Finally, in multivariate analyses, we determined the most significant predictors of smoking and drinking. 

## 2. Materials and Methods

### 2.1. Participants and Procedure

Our study involved 276 students (boys: 54.7%). The sample included adolescents from grade 5 to grade 12 (N = 276), representing the age range of 11–18 years (M = 13.6 years, SD = 1.8). Only five children declined to participate in the survey, which resulted in a response rate of 98%. Data collection was based on convenience sampling in Budapest and villages in its metropolitan area (Hungary). According to the students’ self-assessed financial situation (the students subjectively evaluated their own socioeconomic status with an SES self-assessment [40] as lower, lower-middle, middle, upper-middle, or upper class), 1.4% belong to the lower class, 6.5% to the lower-middle class, 48.2% to the middle class, 37.3% to the upper-middle class, and 6.5% to the upper class. In terms of family background, 62.2% of the participants live with both parents, 16.4% live with one parent and a foster parent, 13.5% live with their mother only, 4% live with their father only, and 4% live with other guardians.

The ethical approval was granted by the Ethics Committee of the Doctoral School of Education, University of Szeged, Hungary. Participation in the study was voluntary and anonymous, and parental informed consent was obtained in all cases. 

The data collection occurred in the school year 2021–2022, using a self-administered paper and pencil questionnaire. The questionnaire took approximately 25–30 min to complete. The researchers, with the help of teachers, made an effort to administer the survey during early school lessons. Thus, the children’s cognitive load was acceptable, and fatigue effects did not affect the results.

### 2.2. Measurements

Beyond socio-demographic data, the questionnaire contained measurements on substance use and its potential protective factors. 

In terms of smoking and alcohol, both lifetime and three-month prevalence rates were obtained. We asked the following questions: “Have you ever drunk alcohol/smoked cigarettes?” and “Did you smoke/drink alcohol in the past three months?” Responses were applied in these analyses in a dichotomous format (No = 0, Yes = 1).

The Hungarian-validated version [41] of the Rosenberg Self-Esteem Scale [8] was applied to measure the students’ global self-worth. The respondents were asked to rate their level of agreement with the statements on a 4-point Likert-type scale (1—strongly disagree; 4—strongly agree). The questionnaire contains 10 items: 5 positive statements (e.g., “I feel that I have a number of good qualities”) and 5 negative statements (e.g., “I feel I do not have much to be proud of”). Higher scores mean greater self-esteem. The reliability of the scale was found to be adequate (Cronbach’s alpha = 0.83).

As a measure of self-control, we used the Self-regulation Scale (SRS) developed by Luszczynska et al. [42]. The questionnaire examines attentional control in cases where individuals pursue their goals despite barriers and setbacks (e.g., “If I am distracted from an activity, I do not have any problem coming back to the topic quickly”). The scale includes 7 items, and respondents are asked to rate on a 4-point Likert-type scale how they feel each statement is typical of them (1—not at all typical; 4—very typical of me). Higher scores indicate higher levels of self-regulation. The scale was reliable, with a Cronbach’s alpha of 0.85 with the current sample.

The Proactive Coping Scale, as part of the Proactive Coping Inventory, was used as a measurement of coping developed by Greenglass et al. [43]. The inventory consists of 7 subscales, of which we used one that contains 14 items. We have chosen this scale because it measures the proactive coping strategies that are relevant to our research. The statements in the questionnaire summarize responses and reactions to specific life situations and ask respondents to indicate on a 4-point Likert scale how they experience the situation (e.g., “I always try to find a way to work around obstacles; nothing really stops me”). The Hungarian-validated version was adapted by Almássy et al. [44]. Higher scores indicate a greater tendency to use this type of coping. The scale shows adequate reliability with the current sample (Cronbach’s alpha = 0.83). 

Resilience was measured using the Hungarian-validated version [45] of the 10-item Connor-Davidson Resilience Scale [46], which measures an individual’s psychological resistance. The items are taken from the original 25-item Connor-Davidson Resilience Scale. Students are asked to decide on a 5-point Likert-type scale how true the provided statements are for them, where 0 is not true at all and 4 is absolutely true (e.g., “I feel like I’m in control of my life”). Higher scores show more resilience. The scale shows adequate reliability with the current sample (Cronbach’s alpha = 0.83).

For school-related resources, we used the Hungarian School Attachment Questionnaire [47]. The 20 items of the questionnaire can be grouped into five factors: general attitudes towards school (6 items, e.g., “I love going to school”); attitudes towards peers (4 items, e.g., “I have lots of friends at school”); attitudes towards teachers (3 items, e.g., “I care about what my teachers think of me”); attitudes towards school subjects (4 items, e.g., “We study few interesting subjects”); and attitudes towards the school environment (3 items, e.g., “I like my schoolyard, there is space to play and relax”). Respondents are asked to rate on a 4-point Likert-type scale how they feel each statement is typical of them (1—not at all typical; 4—very typical of me). Higher scores indicate greater attachment. The subscales were reliable with the following Cronbach alphas: attitude towards school (α = 0.74); attitude towards peers (α = 0.75); attitude towards teachers (α = 0.54); attitude towards school subjects (α = 0.69); and attitude towards the school environment (α = 0.52). These values are quite similar to those in the original study [47]. 

The students’ social support was explored by using the Multidimensional Scale of Perceived Social Support (MSPSS) [48], the Hungarian-validated version [49]. The questionnaire contains three subscales: family (4 items, e.g., “I can talk about my problems with my family”), friends (3 items, e.g., “I can talk about my problems with my friends”), and significant others (3 items, e.g., “There is a special person in my life who cares about my feelings”). In the 10-item questionnaire, students are asked to indicate how strongly they agree with each statement on a 5-point Likert-type scale. Higher scores suggest more social support. These subscales were reliable with the following Cronbach alphas: family support (α = 0.87); friend support (α = 0.88); and significant other’s support (α = 0.80).

In terms of psychological well-being, we used the EPOCH (Engagement, Perseverance, Optimism, Connectedness, and Happiness)—Adolescent Psychological Well-being Questionnaire [50], which assesses five aspects of well-being that together support a higher level of well-being. The Hungarian-validated version was used, which was developed by Láng [51]. Responses for the 20-item, 5-point Likert scale varied from “almost never” to “almost always”. Higher scores indicate a greater level of psychological well-being. The scale was reliable, with a Cronbach alpha of 0.90.

The 5-item version of the WHO Well-Being Questionnaire (WBI-5) [52] was used to measure general well-being, the Hungarian-validated version [53]. The questionnaire includes five statements about the respondents’ feelings over the past two weeks (e.g., “I have felt calm and relaxed”). Responses were measured on a 4-point Likert-type scale (0—at no time; 3—all of the time). Higher scores reflect a greater level of well-being. The reliability coefficient (Cronbach alpha) was α = 0.70 with this sample.

Finally, the Hungarian-validated version [54] of the Satisfaction with Life Scale (SWLS) [37] was used to measure the level of life satisfaction as a general measure of subjective well-being. The students indicated how strongly they agreed with each of the five items (e.g., “I am satisfied with my life), and responses ranged from 1 = strongly disagree to 7 = strongly agree. The final scale had a range of 5–35, where a higher score indicated a greater level of life satisfaction. Although the scale was originally developed for adults, it is now widely used in adolescent populations as well. The scale was reliable, with a Cronbach’s alpha of 0.81 with the current sample. 

### 2.3. Data Analysis

The analyses were completed using the IBM Statistics 25 software package. First, before executing statistical analyses, the rate of missing data was taken into account. Those who did not submit questionnaires were removed from the database. As we checked, the non-participating children (N = 5) did not differ from the overall sample on key characteristics (sex, age, and SES). In a small number of cases, a wholly random instance of missing data occurred, i.e., a single item was absent. These data gaps are random and lack an identifiable pattern. In these cases, we replaced the missing values with 999. Although we cannot find a certain criterion about the acceptable rate of missing data, it is suggested that a missing rate of 5% or less is inconsequential [55]. Thus, we did not need to apply any other imputation method (in our study, the missing values were rare; the rate was around 1–2%, with a maximum rate of 4% in some exceptional cases). SPSS treated these values as missing values (listwise deletion). On this basis, the available case analysis method was employed, i.e., each variable’s entire data set was analyzed. If the number of items in the variable has changed as an outcome, this is indicated when the results are reported. First, descriptive statistics (Student *t*-tests and Chi-square tests) were used to detect group differences. Subsequently, we calculated correlation coefficients for bivariate associations for the scales. Then we implemented bivariate (binary) logistic regression analyses at the 95% probability level to determine the effect of each independent variable on increasing or reducing the odds of substance use. An odds ratio (OR) > 1.0 indicates that there is a positive association between the factors of interest and the baseline odds, while a value < 1.0 indicates the opposite. A maximum *p*-value of 0.05 was used to define statistical significance, and 95% confidence intervals were also calculated for this reason. Finally, multivariate logistic regression was used to determine the most relevant contributors.

## 3. Results

Table 1 shows descriptive statistics for substance use variables (lifetime and three-month prevalence of smoking and alcohol consumption) by sex. 

Frequencies show that 22.3% of the students have smoked in their lifetime and 15.6% have smoked in the last three months, while 56.4% of students have drunk alcohol in their lifetime and 50.7% have drunk alcohol in the last three months. There were no sex differences in the prevalence data (*p* > 0.05).

Table 2 presents descriptive statistics for the scales by sex using *t*-tests for significance.

Boys scored significantly higher than girls on the scales of life satisfaction [t(266) = 1.97, *p* < 0.05] and general well-being [t(268) = 3.22, *p* < 0.001]. Furthermore, girls showed significantly higher scores on one dimension of school attachment, namely, the general attitude towards school [t(272) = −2.60, *p* < 0.01].

Table 3 presents descriptive statistics for the scales as independent variables by current substance user status using *t*-tests for significance. For smoking, we found that there were significant differences between the two groups (smokers vs. nonsmokers) in most variables. Smokers reported lower levels of self-esteem [t(268) = 3.43, *p* < 0.001], self-control [t(268) = 3.10, *p* < 0.01], and family support [t(273) = 4.18, *p* < 0.001]. Among school-related protective factors, attachment to the school environment [t(272) = 2.19, *p* < 0.05], attachment to peers [t(273) = 2.36, *p* < 0.05], and attachment to teachers [t(273) = 2.06, *p* < 0.05] proved to be lower among smokers than nonsmokers. All dimensions of quality of life showed higher scores in smokers than nonsmokers (*p* < 0.001). 

For drinking, we found similar results regarding quality of life measures (*p* < 0.001). Among the individual-level factors, in addition to self-esteem and self-control, lower scores on resilience were also reported by those who drank alcohol [t(266) = 2.36, *p* < 0.05], and they received less social support from significant others [t(274) = 2.22, *p* < 0.05]. In addition, all types of attachment to school showed lower scores among those who consumed alcohol during the past 3 months.

We calculated correlation coefficients for bivariate relationships between the applied scales (Table 4). 

In addition to all the significant correlations, we should highlight some important and strong relationships. Self-control was strongly correlated with resilience [r (259) = 0.67, *p* < 0.001] and social support from the family [r (270) = 0.62, *p* < 0.001]. Family support was also significantly associated with proactive coping [r (257) = 0.53, *p* < 0.001] and resilience [r (263) = 0.59, *p* < 0.001], as well as life satisfaction [r (268) = 0.68, *p* < 0.001], general well-being [r (265) = 0.66, *p* < 0.001], and psychological well-being [r (275) = 0.89, *p* < 0.001]. 

Table 5 and Table 6 show the results of simple binary logistic regression analyses (odds ratios and 95% confidence intervals) for smoking and drinking (lifetime and three-month prevalence). 

Age elevated the risk of experimentation with both smoking and drinking.

In the case of having ever smoked, we found that self-control (OR = 0.90; 95% CI = 0.84–0.96, *p* < 0.001), self-esteem (OR = 0.91; 95% CI = 0.86–0.96, *p* < 0.01), and resilience (OR = 0.95; 95% CI = 0.91–0.99, *p* < 0.01) reduced the chance of trying out smoking. For school attachment, the odds-reducing effect is observed for all dimensions of this variable, including attachment to school, school subjects, school environment, peers, and teachers. This was also the case in terms of quality of life factors. Regarding social support, family support (OR = 0.43; 95% CI = 0.30–0.62, *p* < 0.001) and support from significant others (OR = 0.58; 95% CI = 0.41–0.82, *p* < 0.01) reduced the odds of smoking. 

In connection with alcohol experimentation, we found that individual factors such as self-control (OR = 0.88; 95% CI = 0.83–0.93, *p* < 0.001), self-esteem (OR = 0.94; 95% CI = 0.91–0.98, *p* < 0.01), and resilience (OR = 0.93; 95% CI = 0.89–0.96, *p* < 0.001) had an odds-reducing effect. In terms of school attachment and quality of life factors, the results were similar to smoking, except for attachment to peers, which had a nonsignificant OR (*p* > 0.05). In terms of social support, similar to smoking, family support (OR = 0.53; 95% CI = 0.38–0.72, *p* < 0.001) and support from significant others (OR = 0.63; 95% CI = 0.44–0.90, *p* < 0.01) reduced the chances of trying alcohol.

Subsequently, we explored which factors have an odds-increasing or odds-reducing effect on current substance use (i.e., three-month prevalence) (Table 6). Similar to the lifetime prevalence of smoking, quality of life factors, social support from family and significant others, and self-control and self-esteem were significant predictors here. Among individual factors, resilience did not increase the odds of current smoking. Among school-related factors, only attachment to the school environment (OR = 0.62; 95% CI = 0.40–0.95, *p* < 0.001) and peers (OR = 0.60; 95% CI = 0.39–0.93, *p* < 0.05) were significant predictors.

For alcohol consumption, significant odds-reducing effects were observed for all dimensions of school attachment and quality of life factors. In addition, we found similar results to having ever drunk alcohol, including self-esteem, self-control, resilience, and social support from family and significant others.

Finally, Table 7 presents the results of the multivariate logistic regression analysis. The goodness of fit was significant in all cases. Age (as a risk factor) and self-control (as a protective factor) proved significant predictors of both smoking and drinking (lifetime and three-month prevalence). In terms of having ever smoked, certain social support variables were significant protective factors: family support (OR = 0.29; 95% CI = 0.09–0.97, *p* < 0.05) and support from significant others (OR = 0.34; 95% CI = 0.16–0.73, *p* < 0.01). Regarding having ever drunk alcohol, in addition to self-control, attachment to the school environment was a protective factor (OR = 0.49; 95% CI = 0.24–0.96, *p* < 0.05). However, friend support acted as a risk factor (OR = 1.64; 95% CI = 1.08–2.66, *p* < 0.001). Current smoking was related to attachment to school subjects (OR = 2.87; 95% CI = 1.21–7.36, *p* < 0.05). Attachment to the school environment was again a predictor of current alcohol use (OR = 0.44; 95% CI = 0.23–0.85, *p* < 0.05). Similar to having ever drunk alcohol, friend support was a risk factor (OR = 1.85; 95% CI = 1.15–2.97, *p* < 0.001).

## 4. Discussion

The purpose of this study was to examine a set of potential protective factors at multiple levels for smoking and drinking in a sample of Hungarian adolescents. According to European and worldwide international research, despite a decreasing tendency of traditional substance use among adolescents, such as smoking and alcohol use, it is still a great concern, particularly in Eastern European nations, including Hungary [4,5,6]. Thus, identifying preventive factors may help reduce the occurrence of these health risk behaviors. We approached the concept of protection from the perspective of teenage mental well-being since adolescent mental health was found to be an important public health priority [7,14]. We also used multiple levels of potential protection. Our findings supported the first hypothesis: there were no statistically significant sex differences in adolescents’ substance use; however, girls reported slightly higher rates for the three-month prevalence of smoking and drinking [6]. Likewise, there were only a few sex differences in adolescents’ levels of protection. Calculating the odds of independent variables, self-control seemed to play a universal protective role against substance use, while age was a general risk factor. In addition, certain other potential protective factors (self-esteem, resilience, social support from family or significant others, several aspects of school attachment, and mental well-being) might also contribute to prevention, while friend support acts as a risk factor for alcohol use [36].

The initial step in our research was to look into the occurrence of substance use and differences between the two sexes among adolescents. Previous research on smoking and alcohol use in the Eastern European region confirmed that both lifetime and three-month prevalence rates are higher than those in the Western European region [4,5,6]. Indeed, around 50% of the teenagers had already tried and used alcohol during the previous three months. Prior studies varied in their sex differences for these outcomes [5,6,10]. We discovered no differences in either lifetime prevalence or three-month prevalence between boys and girls. This finding confirms previous research results showing that sex disparities in substance use appear to be decreasing [4,5,6]. Furthermore, girls reported a slightly greater occurrence. Among the protective variables, only a few of them showed sex differences: girls scored lower on the life satisfaction and general well-being scales. This study validates prior research results showing girls’ perceptions of the world during adolescence differ from boys. Adolescent girls may be more sensitive and perceive their world in a different way from numerous viewpoints than adolescent boys, and these perceptions may be more complex and, in many cases, more negative. This represents girls’ increased sensitivity at this age, but it may also result in a higher risk of behavioral and emotional problems [56]. The disappearing sex difference or the slightly higher rate of girls’ substance use compared to boys may also reflect this situation.

We also compared levels of protective factors by substance use based on the current prevalence of smoking and alcohol use. We found differences in various domains between substance users and non-users for both types of substance use. Consumers among the sampled adolescents scored lower, particularly on the following scales: well-being, self-esteem, self-control, and social support from family. These findings support prior research findings that there is a close connection between substance use and certain intrapersonal traits, such as self-esteem and self-control [9,10,11,12,16], well-being [7,39], and family support [35]. Substance use may deplete personal resources, or lower levels of these resources may be a risk factor for substance use through a self-medication strategy. Differences in areas of school attachment also showed their importance in teenage substance use and confirmed prior research findings [28,31,32,33]. Those who were disengaged from school were more vulnerable to being engaged in substance abuse activities, while youth who avoided using substances reported better degrees of school attachment. This finding can point to the protective role of school attachment. Adolescents with positive attitudes toward their school are more involved with school, their teachers, peers, and academics, and they are more likely to engage in healthy behaviors. Our findings suggest that school attachment is particularly relevant in the case of alcohol use.

A similar picture can be seen in the results of bivariate logistic regression analyses regarding adolescents’ self-control and self-esteem, their well-being, and social support from family and significant others. These results confirm prior findings that self-esteem and self-control can have a risk-reducing effect on substance use. These protective factors, together with other personal resources such as self-efficacy, self-image, and effective self-monitoring and planning, can protect against negative health impacts [21,22]. Resilience has been identified as a clear protective factor for the lifetime prevalence of both types of substance use and the three-month prevalence of alcohol use. Namely, adolescents with greater resilience are more likely to say ‘no’ to the temptations of substance use, especially in the initiation stage. This validates prior results showing that resilient adolescents are better at adapting to stressful situations since their resilience may prevent them from engaging in experimentation and regular use of substances [26,27]. From the results, it seems that proactive coping cannot serve as a protective factor; further research is needed to identify more effective coping strategies against adolescent substance use. Personal resources, together with well-being variables, are important protective factors, suggesting that a well-adapted and mentally well-balanced teenager is able to resist health risk behaviors [36,38]. At the interpersonal level, social support from the family and significant others, together with a successful school attachment, also provides protection for adolescents [28,31,32,34,35]. Parental emotional support as well as the parents’ guarding behavior or joint programs together as a family are all relevant aspects of family support [34]. Furthermore, the adolescents’ commitment to their school as well as the quality of their social interactions with teachers and their acceptance of the school’s learning environment can also prevent them from adopting health risks and problem behaviors [28,31,32]. Not surprisingly, age was identified as a risk factor in all cases, while sex did not play a role.

As multivariate analysis suggests, self-control is a universal protective factor against adolescent substance use. On the other hand, age is a universal risk factor, while friend support elevates the risk of alcohol use [36,38]. Peer pressure is a strong determinant of drinking since it is a social activity usually shared with friends. Interestingly, attachment to peers in school, that is, learning together with them, was not a risk factor, while the role of close friends (i.e., the best friend effect in substance use) might differ from this connection [57]. Social support from family and significant others provides protection against smoking, while school attachment seems to be a more important protective factor against drinking. 

While using a complex model of protective factors at different levels is the strength of our paper, we should also mention some limitations. These are the cross-sectional design of our study and the non-representative sampling, which may restrict the generalizability of the findings. Due to the relatively small sample size, we should consider this sample as a first step in a larger study. In addition, the reliability of certain scales (the Hungarian School Attachment Questionnaire) proved relatively low. We did not want to skip them due to their relevance, but this needs further adaptation. All in all, we justified a set of relevant protective factors at multiple levels, which can be target points in a school’s health education program and school curriculum. 

## 5. Conclusions

Overall, the results of this study highlight some important protective factors against adolescent substance use; therefore, they will be valuable for teachers, school psychologists, social and health professionals, and anyone living or working with teenagers. We believe that integrating well-being and related domains into systemic prevention, particularly in school settings, is essential. There is also a need to develop interventions involving families. We really think that a complex prevention program can be the most effective in reducing risk behaviors, promoting preventive health behaviors, and ultimately strengthening mental well-being among adolescents. Based on our results, such interventions should be based on (a) improving youth’s self-control, (b) helping develop school attachment, (c) offering examples for youth on how to socialize with peers without using substances, and (d) building a social network between school and families. 

However, before the development of practical interventions, a solid theoretical framework should be considered. Positive psychology seems plausible for developing a curriculum to foster adolescents’ well-being since it applies a strength model instead of concentrating on pathology and problem behavior [58]. As a positive psychological framework, PERMA (positive emotions, engagement, relationships, meaning, and accomplishments) is suitable for a school environment, providing a strong basis for adopting a positive mindset in both teachers and children [59]. In a study of Australian high school students, a school-based mental health program combining acceptance and commitment therapy and positive psychology was introduced [60]. Using a randomized controlled trial, increased well-being and reduced anxiety levels could be measured as a result of an 8-hour workshop series. In addition to concrete psychological training, however, tools of positive education would also be favorable, e.g., strengthening school communities, forming friendly school environments, and democratizing teacher-student relationships. 

## Figures and Tables

**Table 1:** Frequencies of smoking and drinking in the whole sample and by sex (n = 276).

Variables	Frequency n (%)	Male n (%)	Female n (%)	Chi-Square Tests
Sex				
Male	151 (54.7)			
Female	125 (45.3)			
Lifetime prevalence of smoking				χ^2^ = 0.04, *p* > 0.05
No	212 (77.7)	115 (77.2)	97 (78.2)	
Yes	61 (22.3)	34 (22.8)	27 (21.8)	
Three-month prevalence of smoking				χ^2^ = 0.71, *p* > 0.05
No	233 (84.4)	130 (86.1)	103 (82.4)	
Yes	43 (15.6)	21 (13.9)	22 (17.6)	
Lifetime prevalence of drinking				χ^2^ = 0.01, *p* > 0.05
No	119 (43.6)	65 (43.6)	54 (43.5)	
Yes	154 (56.4)	84 (56.4)	70 (56.5)	
Three-month prevalence of drinking				χ^2^ = 0.39, *p* > 0.05
No	136 (49.3)	77 (51.0)	59 (47.2)	
Yes	140 (50.7)	74 (49.0)	66 (52.8)	

**Table 2:** Descriptive statistics for the scales by sex.

	Male	Female	
Variables	*Mean ± SD*	*Mean ± SD*	t-Value
**Factors of individual protection (Psychological assets)**			
Self-esteem	29.33 ± 6.16	26.06 ± 5.83	4.37
Self-control	20.09 ± 4.41	18.80 ± 4.90	2.26
Proactive coping	42.03 ± 6.99	39.24 ± 6.58	3.28
Resilience	28.24 ± 6.82	25.63 ± 7.31	2.99
**Factors of school-level protection (School attachment)**			
Attachment to school	2.63 ± 0.66	2.84 ± 0.69	−2.60 **
Attachment to school subjects	2.63 ± 0.70	2.67 ± 0.75	−0.50
Attachment to the environment	2.63 ± 0.73	2.74 ± 0.75	−1.15
Attachment to peers	3.27 ± 0.66	3.16 ± 0.73	1.35
Attachment to teachers	3.08 ± 0.71	3.04 ± 0.72	0.49
**Factors of social protection (Social support)**			
Family support	3.75 ± 0.77	3.58 ± 0.90	1.70
Friend support	3.95 ± 1.10	4.15 ± 0.96	−1.63
Significant others	4.42 ± 0.77	4.59 ± 0.71	−1.85
**Factors of mental protection (Quality of life)**			
Satisfaction with life	25.34 ± 6.84	23.67 ± 7.06	1.97 *
General well-being	8.76 ± 2.98	7.54 ± 3.11	3.22 ***
Psychological well-being	3.90 ± 0.58	3.77 ± 0.67	1.78

Notes: Student *t*-tests: * *p* < 0.05; ** *p* < 0.01; *** *p* < 0.001.

**Table 3:** Descriptive statistics for psychological variables by current substance user status.

	Non-Smokers	Current Smokers		Non-Drinkers	Current Drinkers	
Variables	*Mean ± SD*	*Mean ± SD*	t-Values	*Mean ± SD*	*Mean ± SD*	t-Values
**Factors of individual protection (Psychological assets)**						
Self-esteem	28.38 ± 6.20	24.71 ± 5.44	3.43 ***	28.75 ± 5.90	26.96 ± 6.41	2.35 *
Self-control	19.87 ± 4.56	17.47 ± 4.85	3.10 **	20.41 ± 4.48	18.64 ± 4.71	3.15 **
Proactive coping	40.93 ± 7.06	39.71 ± 6.14	1.01	40.82 ± 6.79	40.67 ± 7.08	0.17
Resilience	27.25 ± 7.13	25.92 ± 7.28	1.09	28.12 ± 7.13	26.06 ± 7.05	2.36 *
**Factors of school-level protection (School attachment)**						
Attachment to school	2.75 ± 0.68	2.57 ± 0.67	1.93	2.87 ± 0.66	2.59 ± 0.68	3.41 ***
Attachment to school subjects	2.68 ± 0.74	2.47 ± 0.57	2.20	2.82 ± 0.69	2.48 ± 0.72	4.01 ***
Attachment to the environment	2.72 ± 0 76	2.45 ± 0.59	2.19 *	2.88 ± 0.70	2.48 ± 0.72	4.62 ***
Attachment to peers	3.26 ± 0.70	2.99 ± 0.63	2.36 **	3.33 ± 0.66	3.11 ± 0.71	2.62 **
Attachment to teachers	3.10 ± 0.69	2.86 ± 0.78	2.06 *	3.18 ± 0.65	2.95 ± 0.75	2.59 **
**Factors of social protection (Social support)**						
Family support	3.76 ± 0.81	3.20 ± 0.81	4.18 ***	3.86 ± 0.79	3.49 ± 0.83	3.77 ***
Friend support	4.06 ± 1.0	3.94 ± 1.10	0.67	4.05 ± 1.00	4.03 ± 1.0	0.14
Significant others	4.53 ± 0.69	4.27 ± 0.98	1.65	4.59 ± 0.57	4.40 ± 0.87	2.22 *
**Factors of mental protection (Quality of life)**						
Satisfaction with life	25.19 ± 6.81	21.24 ± 7.03	3.40 ***	26.07 ± 6.82	23.18 ± 6.86	3.45 ***
General well-being	8.49 ± 3.01	6.61 ± 3.09	3.66 ***	8.90 ± 2.89	7.54 ± 3.15	3.65 ***
Psychological well-being	3.90 ± 0.61	3.51 ± 0.64	3.79 ***	3.97 ± 0.58	3.71 ± 0.64	3.53 ***

Notes: Student *t*-tests: * *p* < 0.05; ** *p* < 0.01; *** *p* < 0.001.

**Table 4:** Correlation matrix of study variables.

	2	3	4	5	6	7	8	9	10	11	12	13	14	15
1. Self-control	0.41 **	0.61 **	0.67 **	0.30 **	0.44 **	0.47 **	0.45 **	0.43 **	0.62 **	0.35 **	0.26 **	0.48 **	0.44 **	0.67 **
2. Self-esteem	-	0.52 **	0.46 **	0.26 **	0.22 **	0.27 **	0.37 **	0.17 **	0.48 **	0.26 **	0.16 *	0.45 **	0.48 **	0.49 **
3. Proactive coping		-	0.67 **	0.27 **	0.33 **	0.34 **	0.37 **	0.31 **	0.53 **	0.25 **	0.18 **	0.44 **	0.41 **	0.61 **
4. Resilience			-	0.28 **	0.37 **	0.47 **	0.45 **	0.39 **	0.59 **	0.31 **	0.29 **	0.48 **	0.47 **	0.70 **
5. Attachm. to school				-	0.59 **	0.42 **	0.37 **	0.41 **	0.40 **	0.18 **	0.17 **	0.26 **	0.40 **	0.42 **
6. Attachm. to subjects					-	0.46 **	0.33 **	0.57 **	0.47 **	0.22 **	0.23 **	0.39 **	0.43 **	0.48 **
7. Attachm. to the environm.						-	0.63 **	0.49 **	0.49 **	0.45 **	0.34 **	0.35 **	0.44 **	0.55 **
8. Attachm. to peers							-	0.48 **	0.55 **	0.53 **	0.35 **	0.38 **	0.50 **	0.54 **
9. Attachm. teachers								-	0.44 **	0.22 **	0.33 **	0.35 **	0.32 **	0.53 **
10. Family support									-	0.36 **	0.30 **	0.68 **	0.66 **	0.89 **
11. Friend support										-	0.56 **	0.27 **	0.25 **	0.45 **
12. Support from others											-	0.30 **	0.19 **	0.46 **
13. Satisfaction with life												-	0.54 **	0.69 **
14. General well-being													-	0.59 **
15. Psychological well-being														-

Note: * *p* < 0.05; ** *p* < 0.01.

**Table 5:** Bivariate logistic regression analysis of lifetime prevalence of smoking and drinking (OR: odds ratio).

	Smoking Ever	Drinking Ever
Predictors	OR (95% CI)	OR (95% CI)
**Socio-demographic variables**		
Age (years)	1.92 (1.58–2.34) ***	2.06 (1.66–2.55) ***
Sex		
Male ^a^	1.00	1.00
Female	1.06 (0.60–1.88)	0.97 (0.60–1.56)
**Factors of individual protection (Psychological assets)**		
Self-control	0.85 (0.80–0.91) ***	0.88 (0.83–0.93) ***
Self-esteem	0.93 (0.88–0.97) **	0.94 (0.91–0.98) **
Proactive coping	0.97 (0.93–1.01)	0.98 (0.95–1.02)
Resilience	0.95 (0.91–0.99) **	0.93(0.89–0.96) ***
**Factors of school-level protection (School attachment)**		
Attachment to school	0.64 (0.42–0.97) *	0.51 (0.35–0.74) ***
Attachment to school subjects	0.55 (0.36–0.84) **	0.49 (0.34–0.69) ***
Attachment to the environment	0.58 (0.39–0.85) **	0.47 (0.33–0.67) ***
Attachment to peers	0.53 (0.35–0.78) ***	0.70 (0.49–1.00)
Attachment to teachers	0.57 (0.38–0.84) **	0.61 (0.43–0.87) **
**Factors of social protection (Social support)**		
Family support	0.43 (0.30–0.62) ***	0.53 (0.38–0.72) ***
Friend support	0.82 (0.63–1.07)	0.91 (0.72–1.15)
Significant others	0.58 (0.41–0.82) **	0.63 (0.44–0.90) **
**Factors of mental protection (Quality of life)**		
Satisfaction with life	0.92 (0.88–0.96) ***	0.93 (0.89–0.96) ***
General well-being	0.80 (0.72–0.89) ***	0.84 (0.77–0.92) ***
Psychological well-being	0.40 (0.25–0.64) ***	0.38 (0.25–0.59) ***

Notes: The odds ratios characterizing the relationship between each independent variable and the dependent variable are derived from logistic regression analyses performed separately. ^a^ reference category; OR: odds ratio; CI: confidence interval. * *p* < 0.05; ** *p* < 0.01; *** *p* < 0.001.

**Table 6:** Bivariate logistic regression analysis of the three-month prevalence of smoking and drinking (OR: odds ratio).

	Smoking in the Past 3 Months	Drinking in the Past 3 Months
Predictors	OR ^b^ (95% CI) ^c^	OR (95% CI)
**Socio-demographic variables**		
Age (years)	1.64 (1.37–1.97) ***	2.17 (1.74–2.70) ***
Sex		
Male ^a^	1.00	1.00
Female	0.76 (0.39–1.45)	0.86 (0.53–1.38)
**Factors of individual protection (Psychological assets)**		
Self-control	0.90 (0.84–0.96) **	0.92 (0.87–0.97) **
Self-esteem	0.91 (0.86–0.96) ***	0.95 (0.92–0.99) *
Proactive coping	0.97 (0.93–1.02)	1.00 (0.99–1.03)
Resilience	0.97 (0.93–1.02)	0.96 (0.93–0.99) *
**Factors of school-level protection (School attachment)**		
Attachment to school	0.68 (0.42–1.09)	0.54 (0.37–0.78) ***
Attachment to school subjects	0.67 (0.42–1.07)	0.50 (0.35–0.72) ***
Attachment to the environment	0.62 (0.40–0.96) *	0.46 (0.32–0.65) ***
Attachment to peers	0.60 (0.39–0.93) *	0.63 (0.44–0.90) **
Attachment to teachers	0.64 (0.41–0.98)	0.64 (0.45–0.90) *
**Factors of social protection (Social support)**		
Family support	0.46 (0.31–0.68) ***	0.57 (0.42–0.77) ***
Friend support	0.90 (0.67–1.22)	0.98 (0.78–1.23)
Significant others	0.67 (0.46–0.98) *	0.69 (0.49–0.96) *
**Factors of mental protection (Quality of life)**		
Satisfaction with life	0.92 (0.88–0.97) ***	0.94 (0.91–0.97) ***
General well-being	0.81 (0.72–0.91) ***	0.86 (0.79– 0.94) ***
Psychological well-being	0.40 (0.24–0.66) ***	0.50 (0.33–0.74) ***

Notes: The odds ratios characterizing the relationship between each independent variable and the dependent variable are derived from logistic regression analyses performed separately. ^a^ reference category; ^b^ OR: odds ratio; ^c^ CI: confidence interval. * *p* < 0.05; ** *p* < 0.01; *** *p* < 0.001.

**Table 7:** Multivariate logistic regression analysis of smoking and drinking (OR: odds ratio).

	Smoking Ever	Drinking Ever	Smoking in the Past 3 Months	Drinking in the Past 3 Months
Predictors	OR (95% CI)	OR (95% CI)	OR (95% CI)	OR (95% CI)
Age (years)	2.37 (1.78–3.17) ***	2.42 (1.82–3.20) ***	1.75 (1.37–2.23) ***	2.21 (1.71–2.87) ***
Self-control	0.75 (0.66–0.87) ***	0.84 (0.75–0.95) **	0.83 (0.72–0.95) **	0.89 (0.80–0.99) *
Attachment to school subjects	-	-	2.87 (1.21–7.36) *	-
Attachment to the school environment	-	0.49 (0.24–0.96) *	-	0.44 (0.23–0.85) *
Family support	0.29 (0.09–0.97) *	-	-	-
Friend support	-	1.64 (1.08–2.66) *	-	1.85 (1.15–2.97) *
Significant others	0.34 (0.16–0.73) **	-	-	-
χ^2^	95.57 ***	107.30 ***	61.81 ***	98.90 ***
df	16	16	16	16
Nagelkerke R2	0.51	0.49	0.40	0.46

Notes: The odds ratios characterizing the relationship between each independent variable and the dependent variable are derived from multivariate logistic regression analyses (only significant variables are shown). OR: odds ratio; CI: confidence interval. * *p* < 0.05; ** *p* < 0.01; *** *p* < 0.001.

## Data Availability

The datasets generated during and/or analysed during the current study are available from the corresponding author on reasonable request.
